# Timeliness of Infectious Diseases Referral and Inappropriate Antibiotic Usage Post-Referral in an Asian Tertiary Hospital

**DOI:** 10.3390/tropicalmed4040137

**Published:** 2019-11-18

**Authors:** Liang En Wee, Aidan Lyanzhiang Tan, Limin Wijaya, Maciej Piotr Chlebicki, Julian Thumboo, Ban Hock Tan

**Affiliations:** 1 Duke-NUS Graduate Medical School, 8 College Road, Singapore 169857, Singapore; 2 Department of Infectious Diseases, Singapore General Hospital, Outram Road, Singapore 169608, Singapore; limin.wijaya@singhealth.com.sg (L.W.); piotr.chlebicki@singhealth.com.sg (M.P.C.); tan.ban.hock@singhealth.com.sg (B.H.T.); 3 Preventive Medicine Residency, National University Health System, 1E Kent Ridge Rd, Singapore 119228, Singapore; aidan_lyanzhiang_tan@nuhs.edu.sg; 4 Department of Rheumatology, Singapore General Hospital, Outram Road, Singapore 169608, Singapore; julian.thumboo@singhealth.com.sg; 5 Health Services Research Unit, Singapore General Hospital, Outram Road, Singapore 169608, Singapore

**Keywords:** infectious diseases consult, outcomes, antibiotic stewardship, early referral

## Abstract

Infectious diseases (ID) specialists advise on complicated infections and are advocates for the interventions of antibiotic stewardship programs (ASP). Early referral to ID specialists has been shown to improve patient outcomes; however, not all referrals to ID specialists are made in a timely fashion. A retrospective cross-sectional study of all referrals to ID specialists in a Singaporean tertiary hospital was conducted from January 2016 to January 2018. The following quality indicators were examined: early referral to ID specialists (within 48 h of admission) and ASP intervention for inappropriate antibiotic usage, even after referral to ID specialists. Chi-square was used for univariate analysis and logistic regression for multivariate analysis. A total of 6490 referrals over the 2-year period were analysed; of those, 36.7% (2384/6490) were from surgical disciplines, 47.0% (3050/6490) were from medical disciplines, 14.2% (922/6490) from haematology/oncology and 2.1% (134/6490) were made to the transplant ID service. Haematology/oncology patients and older patients (aged ≥ 60 years) had lower odds of early referral to ID specialists but higher odds of subsequent ASP intervention for inappropriate antibiotic usage, despite prior referral to an ID specialist. Elderly patients and haematology/oncology patients can be referred to ID specialists earlier and their antimicrobial regimens further optimised, perhaps by fostering closer cooperation between ID specialists and primary physicians.

## 1. Introduction

As infectious disease (ID) practice evolves, understanding the frequency and variety of patients referred to ID specialists is important to deliver services, plan training and set research priorities. Trained ID physicians play key roles in coordinating care and providing clinical input on the optimum management of patients with various infections. Multiple studies have demonstrated the value of inpatient referral to ID specialists in improving clinical outcomes in specific syndromes (for instance, *Staphylococcus Aureus* bacteraemia, candidaemia and infective endocarditis) [1,2,3,4] and certain groups of vulnerable patients, such as the immunocompromised host [3,5,6,7]. In particular, early referral to ID specialists within 48 h of admission has been demonstrated to improve patient outcomes and reduce length of stay [7]. However, not all patients are referred to ID specialists early by primary physicians; in studies involving immunocompromised hosts, only about two-thirds of referrals to ID specialists were made within 48 h of admission [7]. Additionally, the bulk of ID inpatient activity is predominantly referral-based [8]; referring physicians have discretion to reject the advice provided. Non-adherence to recommendations made by ID specialists can thus result in inappropriate antibiotic usage. Previous studies have investigated factors associated with adherence to recommendations made by ID specialists [9,10]. Generally, adherence rates to recommendations made by ID specialists are high (~80–90%) [9,10], demonstrating wide acceptability of ID input amongst primary physicians who are prepared to refer their patients to ID specialists. However, acceptance rates vary depending on the type of patient and infection. For instance, acceptance rates are higher in medical compared to surgical disciplines and for nosocomial infections compared to community-acquired infections [9,10]. The mode in which ID specialists’ recommendations are communicated also impacts potential acceptance [9]. The majority of these studies examining acceptance, however, have been conducted in Western societies [9,10]. Given that cultural norms, expectations and modes of communication all play a role in influencing antimicrobial prescribing [11], these findings may not be immediately generalizable to other cultural contexts. While studies from Asian communities demonstrate the value of referral to ID specialists, these studies were generally done in the context of mandatory referrals to ID specialists for specific conditions, and not in situations where referral was voluntary [2,4]. There is a dearth of information in the literature regarding quality indicators of referral to ID specialists, such as timeliness and occurrence of inappropriate antibiotic usage post-ID referral, in the setting of Asian communities.

Singapore is an example of a multi-ethnic, urbanised Asian society. The bulk of tertiary healthcare services are delivered through public hospitals; ID specialist referral services are available in all public hospitals. Previous studies suggested that a high volume of referrals to ID specialists in our local setting came from general medicine and orthopaedics [12]. Immunosuppressed patients formed close to half of all referrals [12]. Given Singapore’s rapidly ageing population and advances in treatments for patients with haematological, oncologic and rheumatological diseases, increasing numbers of immunocompromised patients present to hospitals with opportunistic infections and could potentially benefit from referral to ID specialists [13]. However, despite widespread antibiotic usage and high prevalence of hospital-acquired infections [14], little is known about factors associated with referral to ID specialists in our local population. Other studies in urbanised Asian societies have also demonstrated improved patient outcomes with the involvement of ID specialists [2,4]. In these studies, though, ID specialist referral was made a mandatory routine for certain specific conditions (candidemia and bacteraemia, respectively [2,4] and was not voluntary. In our local setting where ID specialist referral was voluntary and initiated at the request of the primary physician, we were interested in understanding the pattern of ID specialist referrals, the timeliness of ID specialist referral (within 48 h of admission), and factors associated with inappropriate antibiotic usage despite concurrent ID specialist referral.

## 2. Materials and Methods

### 2.1. Study Setting

Singapore General Hospital is a 1785-bed public tertiary hospital in Singapore, with a wide range of specialist services, including stem cell transplant and solid organ transplant programmes. Our centre also has medical, surgical and cardiothoracic intensive care units, as well as a burns unit. In our institution (Singapore General Hospital), ID specialist referrals are replied to by a dedicated team from the Department of Infectious Diseases. The consultation service is available 5 days a week, during working hours; an on-call roster is also available to attend to urgent consultation requests after working hours. The referral service is roster-based; the number of ID physicians on consultation service is available through the hospital operator. Coverage is provided by both a resident and a board-certified specialist (both working hours, as well as overnight/weekend coverage). Recommendations given by the ID resident are always supervised by a board-certified ID specialist. On average, the service receives about 8 new referrals daily. Requests for ID specialist consultation involve the creation of a memo in the patient’s electronic medical record setting out the situation, background and reason for specialist referral to ID; the memo can then be accessed by the ID physician replying to the request for consultation. All new referrals to the ID department go through the standardised process of having a full history taken, bedside physical examination, chart review and a formal reply documented in the patient’s electronic medical record. Referrals to ID specialists are fully voluntary and it is the prerogative of the primary physician to initiate a request for ID specialist referral.

### 2.2. Ethics Approval

The SingHealth Centralised Institutional Review Board approved this study (CIRB Ref: 2017/2631). Only anonymised data were analysed based on data collected as part of routine clinical practice and thus informed consent was not obtained from individual patients.

### 2.3. Study Design

We conducted a retrospective cross-sectional study of all new referrals to infectious diseases in our institution (Singapore General Hospital, a 1785-bed, acute tertiary-care hospital in Singapore), from January 2016–2018. We obtained the following information from SGH enterprise data management systems: patients’ gender, age, comorbidity burden (as measured by the Charlson Comorbidity Index, CCMI), year of referral, admitting location at time of referral (whether in high-dependency/intensive care, versus being in the general ward), whether the patient needed isolation, referring discipline, whether the referral was marked as urgent, and the identity of the replying physician (ID resident or board-certified ID physician). We grouped the referring disciplines into the following groups: medical, surgical, haematology/oncology, and transplant patients. We excluded transplant ID referrals from further analysis because the numbers were small (only 2.1% of all referrals) and because it was felt that these complex patients might not be fully comparable with general ID specialist referrals. Amongst all general ID specialist referrals, we examined the following quality indicators: early ID specialist referral (defined as referring disciplines putting in a request for ID specialist consultation within 48 h of admission) and requiring the intervention of an antibiotic stewardship program (ASP) for inappropriate antibiotic usage during the admission, despite concurrent involvement of an ID specialist. The objective of choosing these two indicators was a) to determine the timeliness of ID specialist referral in our institution and the factors associated with early ID specialist referral, given that early ID referral has been associated with improved patient outcomes and b) to obtain a gauge of adherence to ID specialist input, by using presence/absence of ASP intervention for inappropriate antibiotic usage as a proxy. ASP activities have been established in our institution since 2008, with prospective audit and feedback forming part of our ASP from inception [15]. The ASP team is multi-disciplinary, comprised of both pharmacists and ID physicians; trained clinical ID pharmacists perform the primary review of cases and make ASP interventions as necessary, with ID physicians playing a supporting role. We selected the subset of interventions that were made for inappropriate antibiotic usage; we did not include interventions made for dosage adjustment and recommendations for further investigations. Inappropriate antibiotic usage was defined as either inappropriate duration or choice of antibiotics, as determined after multi-disciplinary review by the ASP team. In our institution, if the patient is already on active review by an ID physician, the ASP team discusses the case with the reviewing ID physician before performing an intervention. Thus, ASP interventions for inappropriate antibiotic usage on patients actively being reviewed by an ID physician are only carried out when both the ASP team and the reviewing ID physician concur that the antibiotic usage is inappropriate.

### 2.4. Statistical Analysis

We computed descriptive statistics for all new referrals to ID specialists, including the percentage that needed concurrent ASP intervention for inappropriate antibiotic usage and the percentage of referrals made within 48 h of admission, stratifying by referring discipline. Subsequently, we identified factors associated with early ID specialist referral (within 48 h of admission) and needing ASP intervention for inappropriate antibiotic usage. We used chi-square or Fisher exact tests, where appropriate, for univariate analysis of categorical variables, and a parsimonious logistic regression model to identify factors that were independently associated on multivariate analysis, using a criteria of *p*-value < 0.1 on univariate analysis as a cut-off for entry of factors into the final multivariate model.

## 3. Results

From 2016–2018, a total of 7245 referrals were made to ID specialists, of which 6490 had complete sociodemographic information (89.6%, 6490/7245) and were further analysed. Of all referrals, 36.7% (2384/6490) were made from surgical disciplines, 47.0% (3050/6490) were made from medical disciplines, 14.2% (922/6490) from haematology/oncology and 2.1% (134/6490) were made to the transplant ID service. The breakdown of referrals by referring disciplines can be found in Table 1. From medical disciplines, the top 3 referring departments were internal medicine, oncology and renal medicine; from surgical disciplines, the top 3 referring disciplines were orthopaedic surgery, general surgery and cardiothoracic surgery. For medical disciplines, the mean time between admission and referral to ID specialists was 6.69 days (standard deviation, S.D= 9.90); for surgical disciplines, it was 8.66 days (S.D 14.03); for haematology/oncology, it was 9.15 days (S.D = 18.03) and for transplant ID referrals, it was 6.31 days (S.D 7.39). Around one-fifth of referrals made to medical disciplines were made within 48 h of admission (17.9%, 545/3050); for surgical disciplines, 18.9% (451/2384) were referred within 48 h of admission, compared with 13.7% (126/922) for haematology/oncology and 39.6% (53/134) for transplant ID referrals. In total, around 18.1% (1175/6490) of referrals were referred within 48 h of admission. One-quarter (23.1%, 213/922) of referrals from haematology/oncology required antibiotic stewardship program (ASP) intervention for inappropriate antibiotic usage despite concurrent referral to an ID physician, compared with 17.7% (423/2384) for surgical disciplines, 13.7% (419/3050) for medical disciplines and 6.0% (8/134) for transplant ID referrals.

Amongst all general ID specialist referrals from 2016–2018 (N = 6356), almost one-fifth (17.7%, 1122/6356) were referred early to ID specialists (within 48 h of admission) and 1055/6356 (16.7%) required subsequent ASP intervention for inappropriate antibiotic usage despite having been seen by an ID physician. The factors associated with early ID specialist referral and subsequent ASP intervention for inappropriate antibiotic usage on univariate analysis are found in Table 2; the factors independently associated with early ID specialist referral and subsequent ASP intervention for inappropriate antibiotic usage on multivariate analysis are found in Table 3. Amongst all ID specialist referrals, on multivariate analysis, patients referred from haematology/oncology had lower odds of referral to ID specialists within 48 h of patient admission (adjusted odds ratio, aOR = 0.69, 95% confidence interval, CI = 0.55 – 0.85) but higher odds of requiring ASP intervention (aOR = 1.44, 95% CI = 1.19–1.73). Older patients (aged ≥ 60 years) had lower odds of being referred to ID specialists within 48 h of admission (aOR = 0.63, 95% CI = 0.55–0.72) but higher odds of requiring ASP intervention (aOR = 1.17, 95% CI = 1.02–1.35).

## 4. Discussion

In our study of referrals to ID specialists, the top referring disciplines were Internal Medicine and Orthopaedics; this is consistent with findings from a 2-week survey of inpatient consultation activities from another Singaporean tertiary hospital, which also identified Internal Medicine and Orthopaedics as the most common sources of referrals [12]. Other surveys of ID specialist referrals in other countries also identified Internal Medicine and Orthopaedics as a common source of referrals [12,16]. However, less than one-fifth of ID specialist referrals were made early (within 48 h of admission), despite findings that early ID specialist referral is associated with improved patient outcomes and reduced length of stay [7]. In particular, patients referred from haematology/oncology had lower odds of early ID specialist referral but higher odds of requiring ASP intervention for inappropriate antibiotic usage. Other studies also suggested that disciplines with traditionally high rates of infectious complications, such as haematology, were surprisingly infrequent users of an ID specialist consultation service [16]. Given that early antibiotic administration is associated with higher survival rates in the context of febrile neutropenia [17] and that having a haematological or oncological condition is associated with high risk of subsequent clinical deterioration and unplanned readmission [7,18], early ID specialist referral should be a part of efforts to ensure that this group of patients receives early and appropriate antibiotic therapy [19]. In local studies evaluating acceptance of ASP recommendations for haematology/oncology patients, acceptance was more likely when the recommendations were made by dedicated ID specialists, rather than a rotating team of specialists or by trainee ID physicians [20], suggesting that early ID specialist referral for haematology/oncology patients could potentially be fostered by building close relationships between haematology/oncology primary physicians and a team of dedicated haematology/oncology ID specialists.

Elderly patients also had lower odds of early ID specialist referral but higher odds of requiring ASP intervention for inappropriate antibiotic usage. The observation that elderly patients had lower odds of early ID specialist referral and higher odds of requiring ASP intervention for inappropriate antibiotic usage was of concern, given that ID specialist referral can reduce mortality and cost of stay in older age groups [21]. Other studies in Asian settings suggested that ID specialist referrals were requested less often for patients with greater disease severity and mortality, many of whom were elderly; patients who received ID specialist consultation conversely had higher rates of alteration to the initially utilised antibiotic [22]. Perhaps the reluctance to refer to ID specialists stems from a concern that ID specialists may disagree with early usage of broad-spectrum antibiotics, especially amongst elderly patients who may present with more severe illness at initial presentation.

In our study, almost one-fifth of patients required subsequent ASP intervention for inappropriate antibiotic usage despite having been seen by an ID physician. This reflects a substantial discordance between primary physicians and ID specialists in antibiotic choice. In other studies, more than one-third of ID specialist referrals already came with a specific antibiotic request from the primary physician [23], suggesting that primary physicians have a pre-determined plan of treatment even when they request an ID specialist consultation. If the ID specialist is seen as the “gatekeeper” for certain broad-spectrum antibiotics [23] and the primary physician has already decided that the patient’s clinical condition warrants usage of such antibiotics, then the ID physician’s input may potentially be ignored when it is discordant with the treatment intention of the primary physician. Multiple reasons have been cited in the literature for non-compliance with restrictive antibiotic prescribing. Physicians may perceive that broad-spectrum coverage may be safer when culture data is lacking or pending [24]. Compliance with restrictive antibiotic prescribing may also be perceived as time-consuming [25]. Additionally, primary physicians may perceive that restrictive antibiotic prescribing impinges on their autonomy [26], particularly when the practices of ID physicians and primary physicians diverge [27,28]. When ID physicians consider restrictive interventions, it is important to adopt a persuasive approach to encourage buy-in [29]. In our centre, there was a statistically significant trend towards early referral to ID in succeeding years, suggesting that closer interaction between ID physicians and primary physicians over time may encourage earlier ID referral.

The limitations of our study were as follows. Significantly, as this was a single-site study, the findings may not be fully comparable to other settings and programs. However, when compared against the findings of our study, similarities exist in the ID case mix of other Singaporean tertiary hospitals [12]. Some of our findings (e.g., disciplines that were the main source of referrals to ID specialists) also concur with previous findings from a 2-week survey of inpatient consultation activities from another Singaporean tertiary hospital [12]. Additionally, this is a cross-sectional study; hence, we were only able to demonstrate associations but not evaluate causality.

In conclusion, there were differences in referral patterns to ID specialists amongst various subspecialties in a Singaporean tertiary hospital. Elderly patients and haematology/oncology patients can be referred to ID specialists earlier and their antimicrobial regimens further optimised, perhaps by fostering closer cooperation between ID specialists and primary physicians.

## Figures and Tables

**Table 1:** Pattern of infectious diseases (ID) specialist referrals in a Singaporean tertiary hospital by disciplines, 2016–2018 (N = 6490).

**Medical Disciplines**					
**Specialty**	**Percentage of Referrals, n%**	**Specialty**	**Days between Admission and Referral (n, S.D)**	**Specialty**	**Required Antibiotic Stewardship Program Intervention within Same Visit (n, %)**
Internal Medicine	1517/6490 (23.4) ^1^	Ophthalmology	2.97 (3.78) ^1^	Oncology	138/605 (22.8) ^1^
Oncology	605/6490 (9.3) ^2^	Dermatology	4.73 (4.68) ^2^	Renal	101/487 (20.7) ^2^
Renal	487/6490 (7.5) ^3^	Endocrinology	6.00 (5.65) ^3^	Haematology	80/391 (20.5) ^3^
**Surgical Disciplines**					
**Specialty**	**Percentage of Referrals, n%**	**Specialty**	**Days between Admission and Referral (n, S.D)**	**Specialty**	**Required Antibiotic Stewardship Program Intervention within Same Visit (n, %)**
Orthopaedic Surgery	551/6490 (8.5) ^1^	Urology	5.27 (4.59) ^1^	Upper GI and Bariatric Surgery	17/47 (36.2) ^1^
General Surgery	455/6490 (7.0) ^2^	Cardiothoracic Surgery	6.41 (5.51) ^2^	Colorectal Surgery	34/120 (28.3) ^2^
Cardiothoracic Surgery	336/6490 (5.2) ^3^	Obstetrics and Gynecology	5.56 (6.51) ^3^	Vascular Surgery	66/290 (22.8) ^3^)

^1, 2, 3^ Numbers in subscripts represent the highest volume of referrals, the shortest time from admission to referral, and the highest proportion of referrals requiring ASP intervention, respectively. We displayed the top 3 disciplines (stratified into medical and surgical disciplines) for each category.

**Table 2:** Demographic and clinical factors associated on univariate analysis with early referral to infectious diseases specialists and requiring antibiotic stewardship intervention despite concurrent infectious diseases specialist referral (N = 6356).

Demographic and Clinical Factors	Early Referral to ID Physician within 48 hrs of Admission, n%	OR (95% CI)	*P*-Value	Antibiotic Stewardship Program Intervention for Inappropriate Antibiotic Usage Despite ID Physician Involvement, n%	OR (95% CI)	*P*-Value
**Gender**						
Female	465/2728 (17.0)	1.00	0.273	462/2728 (16.9)	1.00	0.540
Male	657/3628 (18.1)	1.08 (0.94–1.23)		593/3628 (16.3)	0.96 (0.84–1.10)	
**Age**						
Age < 60 years	563/2459 (22.9)	1.00	<0.001	380/2459 (15.5)	1.00	0.053
Age ≥ 60 years	559/3896 (14.3)	0.56 (0.50–0.64)		675/3896 (17.3)	1.15 (0.99–1.32)	
**Charlson Comorbidity Score (CCMI)**						
CCMI ≥ 5	672/3006 (22.4)	1.00	<0.001	428/3006 (14.2)	1.00	<0.001
CCMI < 5	450/3350 (13.4)	0.54 (0.47–0.62)		627/3350 (18.7)	1.39 (1.21–1.59)	
**Year of admission**						
2016	476/2912 (16.3)	1.00		487/2912 (16.7)	1.00	
2017	510/2790 (18.3)	1.15 (1.00–1.31)	0.054	459/2790 (16.5)	0.98 (0.85–1.13)	0.981
2018	136/654 (20.8)	1.34 (1.09–1.66)	0.007	109/654 (16.7)	0.99 (0.79–1.25)	0.996
**Admitting location at time of referral**						
General ward	1055/5917 (17.8)	1.00	0.194	970/5917 (16.4)	1.00	0.111
Intensive care unit or high-dependency unit	67/439 (15.3)	0.83 (0.64–1.09)		85/439 (19.4)	1.23 (0.96–1.56)	
**Requiring isolation at time of referral**						
No	997/5867 (17.0)	1.00	<0.001	944/5867 (16.1)	1.00	<0.001
Yes	125/489 (25.6)	1.68 (1.35–2.08)		111/489 (22.7)	1.53 (1.23–1.91)	
**Referring service**						
Surgical discipline	451/2384 (18.9)	1.00		423/2384 (17.7)	1.00	
Medical discipline	545/3050 (17.9)	0.93 (0.81–1.07)	0.321	419/3050 (13.7)	0.74 (0.64–0.86)	<0.001
Hematology/Oncology	126/922 (13.7)	0.68 (0.55–0.84)	<0.001	213/922 (23.1)	1.40 (1.16–1.68)	<0.001
**Referral to ID marked as urgent at time of referral**						
No	NA			1027/6258 (16.4)	1.00	0.002
Yes	NA	NA	NA	28/98 (28.6)	2.04 (1.31–3.17)	
**ID referral replied by**						
Resident (under supervision)	783/4150 (18.9)	1.00	<0.001	710/4150 (17.1)	1.00	0.137
Consultant	339/2206 (15.4)	0.78 (0.68–0.90)		345/2206 (15.6)	0.90 (0.78–1.03)	

**Table 3:** Demographic and clinical factors independently associated on multivariate analysis with early referral to infectious diseases specialists and requiring antibiotics stewardship intervention despite concurrent infectious diseases specialist referral (N = 6356).

**Antibiotic Stewardship Program Intervention still Required for Inappropriate Antibiotic Usage Despite Involvement of ID Physician ^1^**	**Adjusted Odds Ratio (aOR), 95% CI**	***P*-value**
Requiring isolation at time of referral (vs. not requiring isolation)	1.56 (1.25–1.96)	<0.001
Referred from medical discipline (vs. referred from surgical discipline)	0.74 (0.64–0.86)	<0.001
Referred from hematology/oncology (vs. referred from surgical discipline)	1.44 (1.19–1.73)	<0.001
Urgent referral to ID (vs. non-urgent referral)	2.05 (1.31–3.20)	0.002
Age ≥ 60 years (vs. age < 60 years)	1.17 (1.02–1.35)	0.025
**Early referral to ID physician within 48 h of admission ^1^**	**Adjusted odds ratio (aOR), 95% CI**	***p*-value**
Requiring isolation at time of referral (vs. not requiring isolation)	1.71 (1.38–2.13)	<0.001
Referred from medical discipline (vs. referred from surgical discipline)	0.92 (0.80–1.06)	0.225
Referred from hematology/oncology (vs. referred from surgical discipline)	0.69 (0.55–0.85)	<0.001
Age ≥ 60 years (vs. age < 60 years)	0.63 (0.55–0.72)	<0.001
Charlson Comorbidity Score < 5 (vs. CCMI ≥ 5)	0.62 (0.54–0.71)	<0.001
Admitted in 2017 (vs. admitted in 2016)	1.13 (0.98–1.30)	0.092
Admitted in 2018 (vs. admitted in 2016)	1.20 (1.05–1.62)	<0.001

^1^ A cutoff of *p* < 0.1 on univariate analysis was used for initial entry of factors into multivariate logistic regression models; the most parsimonious model was then derived via stepwise removal of variables that did not meet the significance criteria of *p* < 0.05 on multivariate analysis.
